# EthniCity of Leisure: A Domains Approach to Ethnic Integration During Free Time Activities

**DOI:** 10.1111/tesg.12307

**Published:** 2018-02-20

**Authors:** Kristiina Kukk, Maarten van Ham, Tiit Tammaru

**Affiliations:** ^1^ Department of Geography University of Tartu, Faculty of Science and Technology, University of Tartu Tartu Estonia; ^2^ OTB – Research for the Built Environment, Faculty of Architecture and the Built Environment Delft University of Technology Delft the Netherlands; ^3^ School of Geography and Sustainable Development University of St Andrews Fife Scotland UK

**Keywords:** Leisure time, ethnic segmentation, ethnic segregation, domains approach, mixed methods, Estonia

## Abstract

This paper investigates the most common leisure time activities, activity sites and the interaction between members of minority and majority populations as they spend their time out‐of‐home and out‐of‐workplace. We ask the question how leisure time activities are related to the ethnic dimensions of residential and workplaces. Our case study area is Tallinn, Estonia, and the main findings show that leisure time activity patterns have become very similar across the main ethnic groups, which is different from what is found for workplace and residential segregation. This shows the integrative potential of leisure time activities. However, since members of the minority and majority population still tend to visit different leisure sites, there is little interaction. We also find that people often spend their free time close to home, which implies that high levels of ethnic residential segregation translate into ethnic segregation during leisure time.

## Introduction

This paper focuses on segmentation and segregation during leisure time. Contemporary urban life offers ample opportunities for out‐of‐home and out‐of‐work leisure time activities, ranging from having a cup of coffee next door with a best friend, to attending mega‐concerts that attract large crowds of people from both the city and outside. Oldenburg (1989) used the term ‘third place’ to highlight the importance of non‐work and non‐home activities, and argued that free time activities are important in building a sense of community. Contemporary cities are also increasingly diverse, providing a shelter for those living in extreme poverty as well as for those enjoying large wealth, people with different ethnic and cultural backgrounds and so on (Vertovec 2007; Piekut *et al*. 2012). Meeting and mutual learning during leisure time could harness the much cherished positive aspects of diversity that pertains to learning and transfer of skills and would contribute, ultimately, to higher levels of ethnic integration in modern cities.

However, more sceptical views exist as well, showing that even so‐called ‘diversity‐seeking’ people show little engagement with local, social or political affairs (Peters & de Haan 2011). For understanding ethnic integration during out‐of‐home and out‐of‐work leisure time, both segmentation and segregation dimensions of free time activities need to be considered. The term segmentation refers to the different out‐of‐home and out‐of‐work activities that people undertake during free time, that is, going out to pubs or doing sports; so different groups might undertake different activities. With segregation we refer to the actual locations of activities, such as the actual pub people go to (Kamenik *et al*. 2015); different groups might undertake the same activities, but in different locations. Overcoming segmentation of free time activities is a pre‐condition for overcoming segregation, which is in turn a precondition for interaction between people from different backgrounds.

The over‐arching goal of this paper is to learn more about the ethnic integration processes during leisure time. More specifically, we will contribute to the existing literature on segregation by focusing on both the segmentation and segregation dimensions in facilitating inter‐ethnic contact during the most common out‐of‐home leisure time activities in Estonia. An important precondition for interactions between ethnic groups is undertaking the same activities at the same place at the same time (Silm & Ahas 2014; Toomet *et al*. 2015). We will analyse both activities and places, down to micro‐public spaces such as sports clubs and pubs, where people can develop intercultural understanding through interaction and exchange around common interests (Amin 2002; Valentine & Sadgrove 2012).

We will take a domains approach developed by van Ham and Tammaru (2016) by seeking what is the role of important other spheres of daily life – residential neighbourhoods and workplaces – in facilitating joint free time activities between ethnic groups. The data for our research was collected in the capital city Tallinn. We used a mixed‐method approach in order to study the segmentation and segregation dimensions of out‐of‐home and out‐of‐work leisure time activities. The Estonian Time Use Survey (2000 and 2010) was used to better understand changes in the segmentation of leisure time activities over time. Qualitative interviews were used to get deeper insights into the links between ethnic segmentation and ethnic segregation during leisure time.

## Ethnic Segmentation, Segregation and Interaction During Leisure Time

The core of the domains approach is to understand the co‐evolution of ethnic integration processes in different spheres of daily life (van Ham & Tammaru 2016). For adults, these spheres mainly include home, work and leisure time. Our specific focus in this paper is on out‐of‐home and out‐of‐work leisure time activities and how they are related to home and work. Departing from Meissner (1971) who characterised leisure as a ‘long arm of work’, we are interested how different domains are linked with each other; with other words, what is the role of both workplaces and residential neighbourhoods in shaping ethnic integration during out‐of‐home and out‐of‐work leisure time. In so doing, we distinguish two underlying dimensions of ethnic separation during leisure time: segmentation and segregation.

*Segmentation* refers to the structure of leisure time activities and how similar or different they are between ethnic groups. Segmentation in leisure activities can emerge as a result of differences in wealth and preferences between ethnic groups (Shinew *et al*. 2004; Kamenik *et al*. 2015). Being able to afford desired leisure activities and socialising with people who have similar values and socio‐economic status has an impact on an individual's feeling of success, and leisure has become an important part of social identity, lifestyle, quality of life and life satisfaction (Roberts *et al*. 2001). Since ethnic minorities tend to niche into less‐skilled labour market segments (Schrover *et al*. 2007), there are differences in leisure time activity patterns compared to the members of majority population both through differences in salaries and status identification (Dutton *et al*. 1994).

Segmentation relates also to preferences that in turn tend to facilitate ethnic enclosure as a result of the interactions with people similar to each other. Many cultural activities tend to separate ethnic groups since, in an ethnically diverse city leisure time often provides members of the minority population with the opportunity to preserve their own cultural and ethnic identity (Floyd & Gramann 1993; Gentin 2011; Kamenik *et al*. 2015; Kim *et al*. 2015). For example, minorities often use leisure time for maintaining and developing deep meaningful relationships with their family and people of the same origin who have similar cultural background and life experiences. Immigrants arrive in their host country with already developed culturally embedded leisure participation preferences and patterns, they celebrate specific holidays, participate in familiar leisure activities, and have similar tastes in music, ways to party, leisure venues, etc. (Kim *et al*. 2015). Celebrating such traditional activities even over several generations is very common in ethnically diverse cities (Silm & Ahas 2014).

*Segregation* refers more explicitly to the spatial dimension of leisure time activities, namely, to the places of encounter. Even when members of the different ethnic groups undertake similar activities, it does not necessarily imply that they meet each other; these activities need to take place both at the same place and at the same time for ethnic interaction to occur (Silm & Ahas 2014; Toomet *et al*. 2015). While leisure time activities are partly related to social and ethnic identities, sorting into different residential neighbourhoods and the consequent high levels of residential segregation potentially affect the choice of leisure time activity sites. Many leisure activities still take place close to home, and the neighbourhood of residence is generally more important in structuring the daily life of ethnic minorities compared to the majority population (van Kempen & Wissink 2014). Hence, leisure can also be characterised as a ‘long arm of home’.

It should be noted that the individual tendency towards homophilious relations (McPherson *et al*. 2001) and discrimination (Schelling 1971) – either direct or indirect – is an important reason for both the segmentation and segregation dimensions of ethnic separation in leisure time activities. Valentine and McDonald (2004) have disclosed that prejudice is being justified with arguments that the other group is not behaving like ‘us’ and that this behaviour is believed to show minorities’ failure to integrate. Behaving differently or not following the ‘behavioural code’ of the majority's culture could be frowned upon by some members of the majority population and possibly even cause direct avoidance of places visited by ethnic minorities (Dixon & Durrheim 2003; Sime & Fox 2014). Valentine (2008) also warns that contemporary cities are often over romanticised as ‘sites of connection’ since the grim reality shows that most everyday contacts are brief and passing, and do not entail deeper social interaction between ethnic groups.

To conclude, it is important to undertake the same leisure activities (no segmentation) at the same places at the same time (no segregation) for inter‐ethnic contacts and interaction to emerge. Following the domains approach, we consider leisure, among other things, as being the ‘long arm of work’ and ‘long arm of home’. Both labour market and residential segregation between ethnic groups tends to be persistently high in European cities (Strömgren *et al*. 2014; Tammaru *et al*. 2016). In this context we ask, what is the role of places of work and residence in ethnic integration during leisure time on the one hand, and whether leisure time activities have the potential to drive ethnic integration on the other hand.

## Case Study: Tallinn

Data for this study comes from Tallinn, the capital of Estonia with about 400,000 inhabitants on a total of 1.3 million people living in Estonia. Of the inhabitants of Tallinn 55% are Estonians and 41% are Russian‐speaking minorities of which Russians form almost 90% (Statistics Estonia 2016). Tallinn was one of the main destinations for immigration when Estonia was part of the Soviet Union (1944–1991). This makes Tallinn a city where two ethno‐linguistic groups are almost equal in size; while Estonian form a clear majority in Estonia as a whole, this is not the case in the capital city.

When Estonia regained independence in 1991, immigration to Estonia stopped. This implies that one important factor that shapes inter‐ethnic relations, the arrival of new immigrants, is eliminated from our case study context from the early 1990s. This creates an interesting experimental ‘laboratory’ setting for investigating the changes in ethnic relations over time. In Estonia, creating inter‐ethnic relationships has been inhibited by the separate language school system for Estonian and Russian‐speaking children, which was created during the Soviet period and is still largely functioning today. Starting from kindergarten, language‐based networks are formed and they are very difficult to breach by inter‐ethnic relationships in adult life.

Previous research shows that both labour market segregation, and especially residential segregation grew in Tallinn between the 2000 and 2011 censuses; minorities are performing worse on the labour market than the majority group, and this is increasingly translated into minorities sorting into the lowest social status neighbourhoods (Tammaru *et al*. 2016). The opportunities to spend leisure time out‐of‐home and out‐of‐employment facilities were limited during the Soviet time; the service sector was under‐developed and there were very few cafés or shopping malls. After Estonia regained independence, the service sector, including leisure time activity sites, started to mushroom in Tallinn like in every other Eastern European city. If leisure serves as the ‘long arm of work’ and ‘long arm of home’, we can expect increasing differences in leisure time activities as well. However, if we find evidence of decreasing leisure time segmentation and segregation, it would imply that leisure time activities have an important integrative potential in today's ethnically diverse cities.

## Data and Methods

We study both the segmentation and segregation dimensions of ethnic differences in leisure activities. In order to capture both of those dimensions, we combine the two last waves of the Estonian Time Use Survey from 2000 and 2010 with qualitative in‐depth interviews. Time use surveys provide us the big picture in the changes of time use by activity (segmentation), and the two waves correspond with census years. This allows us to compare changes in leisure time activities with changes on the labour market and changes in residential segregation. The in‐depth interviews help us to tease out to what extent leisure time activities are taking place at the same place (segregation) and whether they involve meaningful contact and interaction.

The Estonian Time Use Survey is conducted by the Estonian Statistical Office following guidelines of the Harmonised European Time Use Surveys by Eurostat. The samples are non‐proportional stratified samples drawn from the Population Register. The selected person brought his/her immediate family (all family members who were at least 10 years old) to the sample. The size of the sample is 6,438 individuals in the 2000 survey and 7,313 individuals in the 2010 survey. We limited our research population to people living in Tallinn and those who are at least 15 years old, which amounted to 1,161 individuals in 2000 and to 810 individuals in 2010. The sample included 54% and 48% of Estonians in 2000 and 2010 respectively, corresponding well to the percentages in the general population.

We applied binary logistic regression on the data, and the dependent variable was coded as follows: 1 – if the individual participated in different leisure activities during the previous year; 0 – if the individual did not participate in different leisure activities during the previous year. In total we constructed 12 models for different out‐of‐home and out‐of‐work leisure activities including culture‐related activities (culture‐related activities in total, attending theatres and concerts, going to cinemas and museums/art galleries), entertainment activities (entertainment activities in total and going to restaurants/pubs, nightclubs, casinos, fun fairs/zoos), spending time in nature and doing sports. The activities in the analysis are of wide variety and include the most common leisure activities taking place outside people's homes and providing a possibility for meeting and socialising with other people, including people from other ethnic groups. Participation rates for every activity are shown in Table 1.

**Table 1:** Participation in leisure time activities by ethnicity (%) in 2000 and 2010 in Tallinn.

	Total		Estonians		Russians	
	2000	2010	2000	2010	2000	2010
Culture	63.3	79.0	73.3	85.4	55.7	75.1
Theatre	42.0	55.0	50.7	62.9	35.1	50.0
Concert	45.9	63.8	55.3	68.0	39.6	61.1
Cinema	27.3	45.1	35.0	48.6	21.3	44.3
Museum/art gallery	34.3	56.7	42.7	63.8	26.5	50.8
Entertainment	64.5	79.5	70.1	83.1	60.7	76.7
Restaurant/pub	47.5	69.3	53.1	75.0	44.8	63.7
Nightclub/disco	24.5	22.1	29.9	26.7	21.0	18.1
Casino	6.8	2.4	6.6	3.3	7.3	1.7
Funfair/zoo	39.8	42.5	43.6	44.1	34.6	42.0
Nature	62.2	85.8	56.0	84.8	70.2	86.5
Sports	66.9	45.7	71.4	50.6	62.7	42.0

*Note*: 2000 N = 1,161; 2010 N = 810

Our main variable of interest in the models is ethnicity. Ethnicity is self‐defined by people and, in the Estonian context, it strongly overlaps with mother tongue. Estonians speak Estonian, and most of the minorities speak Russian as their mother tongue. We include only Estonians and Russians (80% of the minorities) into our final research population. We include several important control variables affecting leisure participation into our models such as sex, age, family's income decile, marital status, labour market status, education and whether a person has a car in their family (cf. Kamenik *et al*. 2015). These variables also allow us to control to what extent ethnic differences in leisure activities are actually due to other personal characteristics, such as socio‐economic status. We only present results on ethnicity since this is where our main interest lies in this paper. Since the data were collected at the household level, we used the survey method in STATA (StataCorp, College Station, TX) with the household as the primary sampling unit, in order to eliminate the effect of clustering of people into households in our regression models.

Using data from the Estonian Time Use Survey enables us to find out whether Estonians and Russians participate in the same type of activities, but it does not give us information whether they go to the same places at the same time, and thus have the possibility to meet and interact with each other. There are no large‐scale representative surveys that would capture both of the dimensions we are interested in, segmentation and segregation, in generating inter‐ethnic contact and interaction. In order to overcome this problem and to also capture the depth of social interaction between ethnic groups, we conducted 24 in‐depth interviews among 11 Estonians, 11 Russians and two people of mixed ethnic origin (Estonian and Russian).

The first contacts for the interviews were made using acquaintances of the research team members who were then asked to bring the researchers into contact with their more distant acquaintances for new interviewees. It was closely monitored that those interviewed were not close friends because of their overlapping networks and similarities in behaviour. In order to guarantee a good spread of respondents, some respondents were recruited from the streets of different districts of Tallinn. Our strategy was to interview people with networks of different sizes and ethnic compositions, people with different workplaces, and people who live in residential neighbourhoods with different ethnic compositions, for example, people who live in districts with an ethnically mixed composition or with one ethnic group being over‐represented. All interviews were conducted in people's native languages and were subsequently translated into Estonian. Only those extracts of the interviews that are used in this paper have been translated into English.

## Results: Decreased Segmentation but Persistent Segregation During Leisure Time

The regression analysis shows that the effect of ethnicity has undergone important changes between the 2000 and 2010 surveys (control variables not shown). In 2000, Estonians had greater odds for participating in all culture activities and in some entertainment activities, while Russians were more likely to spend time in nature. Ethnically neutral activities were going to the casino and visiting a restaurant or pub. Ethnic difference in leisure time activities almost disappeared by 2010 (Table 2), with the only exception being going to restaurants or pubs. Immigration to Estonia stopped in 1991 after regaining independence from the Soviet Union, while most of the Soviet time migrants and their children remained Estonia. In 2000, after the first decade of socio‐economic transformations, Russians in Estonia were clearly hunkering down and isolating themselves within their home environments and co‐ethnic networks. A middle‐aged Russian woman who became unemployed as a result of economic restructuring in the 1990s explained: ‘Because of their hard lives people changed, they used to be much kinder and open [during the Soviet time]’. However, as the first important finding we see that with time, ethnic Russian participation in most of out‐of‐home and out‐of‐work leisure activities has become equal to that of Estonians, despite the fact that they perform less well on the labour market compared to Estonians and that they are becoming more and more residentially segregated (cf. Tammaru *et al*. 2016).

**Table 2:** Participating in out‐of‐home leisure time activities in Tallinn 2000 and 2010 (odds ratios, ref. Russian).

Leisure activities	2000	2010
Culture total	2.72^***^	1.33
Theatre	1.77^***^	1.10
Concert	1.87^***^	0.86
Cinema	2.34^***^	0.74
Museum/art gallery	1.85^***^	1.13
Entertainment total	1.64^***^	1.39
Restaurant/pub	1.22	1.45^*^
Nightclub/disko	1.56^**^	1.51
Casino	0.99	1.14
Fun fair/zoo	1.73^***^	1.13
Nature	0.47^***^	0.82
Sports	1.63^***^	1.08

*Notes*: ***p < 0.01, **p < 0.05, *p < 0.1. 2000 N = 1,161; 2010 N = 810

The qualitative interviews convey a similar message: Estonians and Russians now participate in similar leisure time activities. After the initial shock related to the dissolution of the Soviet Union and the cessation of immigration as a result, and the increased wellbeing of people especially since Estonia joined the European Union in 2004, there is less ‘hunkering down’ behaviour. Both Estonians and Russians are actively spending more of their leisure time out‐of‐home and out‐of‐of‐work, and their free time activities have become very similar. But our interest in the interviews lies elsewhere: to tease out what are the activities that bring together ethnic groups, whether they bring along deeper social interaction between members of the minority and majority populations, and whether the location of homes and workplaces shapes the choice of the free time activity sites. We find that although the activities of Estonians and Russians have become very similar, this does not necessarily mean that Estonians and Russians meet and interact with each other. The differences in the ethnic geography of leisure pertain both to the larger‐scale spatial units such as city districts, as well as smaller‐scale differences such as specific places one or the other group prefers to visit, down to micro differences at the level of activity site itself.

Despite the convergence of leisure activities, Estonians and Russians do not often meet each other during free time. Leisure time activities are social activities, which implies that social networks still operate mainly among ethnic groups. Many of the interviewees talk about close relationships with old friends from high school or university, current and old work colleagues, or others with whom they share similar interests or life experiences. In Tallinn, almost half of the population is formed by a mainly Russian‐speaking minority that arrived between 1944 and 1991 and there has been very little new immigration. Hence, despite the fact that the schoolmates, colleagues and friends of Russians also live mainly in Estonia, the social networks of Estonians and Russians do not overlap very much, and hence the social activities during free time, although being similar, tend to be ethnically homophilious and take place at different places at different times.

Interestingly, the place of residence rather than the place of work is often related to choice of free time activity sites. To start with, we find that many interviewees have friends or relatives living nearby. It seems that living close to friends of friends often happens by chance as similar people make similar choices when they are looking for a home. An experience shared by a 29‐year‐old Estonian male, working as a book printer:

One of the friends of my close friend is my next‐door neighbour. That was a pleasant surprise. But yes, Jaanus lives maybe 200 metres from here, then Toomas lives just across the railway. Siim lives here, Annika lives here, my ex‐girlfriend's friend lives here. And then of course, I know people who live somewhere in the neighbourhood but have not visited yet.

This finding seems to be in contrast to many views of today's society such as the new mobilities paradigm (Sheller & Urry 2006) or ‘community liberated’ (Wellman 1996). We do not question the evidence of growing mobility, including residential mobility, experienced by the residents of Tallinn, our case study city (cf. Mägi *et al*. 2016). However, our findings do show that these views tend to downplay the importance of residential neighbourhoods in the daily life of people, at least when it comes to the shaping of social networks and out‐of‐home and out‐of‐work leisure time activities. Furthermore our findings are in line with some other earlier studies (Clayton 2012; Schaeffer 2013). In other words, while the daily interaction with neighbours is often superficial, home is still an important anchor point (see Mooses *et al*. 2016) of our daily activities and hence it has an important effect on other places we visit. Hence, leisure could be partly considered as a ‘long arm of home’.

Ethnic residential segregation is growing in Tallinn, partly because labour market outcomes are very different between ethnic groups (Tammaru *et al*. 2016). This is in line with what was found across Europe; there is increasing evidence of a growing overlap between social and ethnic segregation (Marcińczak *et al*. 2016). The potential for establishing vertical weak ties across social and ethnic groups in neighbourhoods is not high, and such ties could rather emerge in the work domain (Ryan 2016). Jackson and Butler (2014) talk about social tectonics to indicate limited ties and interactions between old and new inhabitants in gentrifying neighbourhoods. We find similar socio‐ethnic tectonic plates in Tallinn (cf. Tammaru *et al*. 2016) as different socio‐ethnic groups sort into different neighbourhoods of the city, which influences the location and type of places people visit in their leisure time. The leisure time activities also reflect the preferences of ethnic groups, as illustrated by one of our interviewees living in a gentrified neighbourhood:

This question – where all the Russians are – has been bothering me for a very long time. I think the Russians are in their own ‘Kalamaja’. I don't want to say that all the Russians live in Õismäe, Lasnamäe, or Kopli [these are all different districts of Tallinn]. But these are their neighbourhoods, that's where their ‘tribe’ is and where they want to be. […] If someone asked me to describe the places Russians go to [during the leisure time], I would say the places are more glamorous, demanding, a little over the top. And the places Estonians like to go to, have more of a vintage, worn‐out ambience, and ironically – Soviet‐style.

The explanations for differences in leisure activities and locations, thus go beyond structural factors as differences in income and residential location, as they are also related to taste and preferences. Although cafés in mixed‐ethnic neighbourhoods are by no means exclusive to certain ethnic groups, the atmosphere and tacit rules of etiquette are still recognisably different. Northern Tallinn is one of the most ethnically diverse districts in Tallinn, and our interviewees point to important ethnic differences when selecting cafés in this part of the city:

Bars in Northern Tallinn are very nice cultural experiences, because life there is different … Maybe it is more of a question of perception because I mostly go to Estonian places … But it seems that there [in Russian bars] are some different rules and those rules are more rigidly fixed – you can feel that there is some kind of etiquette and you perceive very strongly how you are expected to behave and what you should not do. (Male 20, Estonian)

The fact that people want to meet people similar to them is probably one of the reasons why different ethnic groups prefer different leisure venues, as this helps to avoid conflicts and discrimination, as also shown by previous research (e.g., Dixon & Durrheim 2003; Valentine & McDonald 2004; Clayton 2009; Harinen *et al*. 2012). Especially in rapidly gentrifying neighbourhoods, Estonians have created new places for themselves, like the Telliskivi Creative Centre with lots of cafés, an open stage for performances, and other leisure time activities, which are seldom visited by Russian speakers. These gentrifiers are often seen as ‘diversity‐seekers’, but we find little evidence that the group of ‘diversity‐seekers’ explicitly look for ethnic diversity or inter‐ethnic interaction. Instead they look for places with a certain character, often cosmopolitan places when it comes to values and consumption rather than local interaction with people of a different kind.

The Estonian‐Russian language divide is especially clear in Tallinn. For example, events in Telliskivi Creative Centre, located in one of the most ethnically diverse districts, tends to be language based: the language of announcements, advertisements, events or instructors often determines whether Estonian speakers or Russian speakers are attracted. Such information about events also appears in different media channels that are either in the Estonian language or in the Russian language. Even the city has two official newspapers, one in Estonian and another one in Russian, and there is little overlap in content. Furthermore, more personalised networking and exchange of information about leisure time events in social media, for example on Facebook, is language based too:

Some people are very active [on Facebook], there is a man who knows everything about the history of Pelgulinn and who posts info about the streets and buildings … They are opening a new Maxima [retail chain in Estonia] somewhere in Northern Tallinn … I would have not even known about it. (Female, Estonian)

People with different ethnic backgrounds do visit the same places too, but this happens often at different times. For example, during the daytime, members of different ethnic groups often lunch together at the same cafés, but this changes in the evening. Evening entertainment activities last longer than daytime lunches, taste in music and the style of places become more important, and this starts to sort Estonians and Russians into different places. Likewise, the social interaction between people is more intimate in the evening, and people choose more carefully where and with whom they spend their evening leisure time.

There is a Georgian restaurant in Lasnamäe where you can go in the daytime and the clientele might be fifty‐fifty by spoken language. But when I happen to go there on Friday evening, I guarantee that 90% of the customers are Russian‐speaking. (Male, 35, Estonian)

A similar pattern of visiting the same activity site but at different times emerged from religious activities.

We have two congregations [in our church], Russian and Estonian. We go to the same building. Russians go there at a different time since their service starts after our service has finished. (Female, 59, Estonian)

We find strong evidence of micro‐level segregation during free time activities within activity sites themselves. Estonians and minorities do spend free time at the same place at the same time, but it is often merely co‐presence without social interaction taking place. Previous studies using quantitative methods have stated that integration takes place when different ethnic groups visit the same leisure sites (Toomet *et al*. 2015). But our results show that the fact that people from two ethnic groups frequent the same places at the same time, does not mean that they really interact and develop meaningful relations. Similar to Kivijärvi (2015), our respondents report that Estonians and Russian‐speakers tend to prefer in‐group contacts even when they are at the same place at the same time. One reason that keeps out‐group communication to a minimum is again the language barrier, which is mentioned by both Estonians and Russian‐speaking interviewees:

When I hear that people are talking in Russian I do not go to speak with them just because of the language barrier. It is awkward to go and start speaking to them in English … although I am a very social person … it is totally leaving my comfort zone and especially when I realise that they do not speak Estonian at all or even conversation in English comes very unnaturally – why should I put myself in such situation? (Male, 25, Estonian)

For me personally, participating in the company's parties is quite difficult because everybody except me and some drivers are Estonians. They all chat together, but I sit with the drivers and talk to them. Sooner or later I get bored and want to leave sooner. (Male, 28, Russian)

The language barrier can also work more indirectly. Even when Russians actually can speak Estonian or Estonians can speak Russian, they feel uncomfortable speaking in a foreign language during their free time when there is an opportunity to speak in their native language. So it is almost inevitable that if there is more than one person of the other ethnic group, communication and friendships are formed based on ethno‐linguistic divisions:

While working out I became friends with Veronika, but she is not Estonian. There were some Estonian girls also, but we did not talk much with each other because all the time they were separate from Veronica and me, we talked more with Russians. (Female, 27, Russian)

Finally, we do find evidence that ethnic divides have decreased, and visiting the same places at the same time can lead to social interaction, especially among the younger generations. This is probably because the younger generation is born in an independent Estonia and there is hardly any new immigration. With time, both Estonians and Russian‐speakers are undergoing changes; prejudices have started to decrease, leisure preferences have become more similar, and the language barriers are starting to diminish when more and more young Russians speak Estonian. Sports are one of the activities where close contacts are relatively easy to form because it is based on common interests (Harinen *et al*. 2012; Kim *et al*. 2015). Our results confirm that common interests, not only in sports, helps to cross inter‐ethnic boundaries in different leisure activities:

A good example is beach volleyball where we have the net and the ball and some people but not enough for playing. Then when you find some other group who wants to play, it is very easy to get new contacts because you have a common interest … We exchange contact details and next time we call them and ask to get the group together and join us. (Male, 25, Estonian).

Kodu Bar is very nice place and I think that the situation is changing and there will be more and more such places … This is a place where cultural or not stereotypical Russian young people go and very many Estonians go there too, and this place has an extremely cool atmosphere where these two cultures very nicely meet. More like based on interest, or on music, or who like's underground lifestyle. (Male, 30, Estonian)

These kinds of ethnically mixed places show that ethnic boundaries are becoming somewhat more blurred in the past ten‐fifteen years, segregation during leisure has decreased and inter‐ethnic interaction is increasing. Young people are far more active during their leisure time than middle‐aged and older people (Kamenik *et al*. 2015). As families are formed, people are much less engaged in the out‐of home free time activities. Estonians are more successful on the labour market than ethnic minorities (Tammaru *et al*. 2016). Estonians use their stronger purchasing power to move into more attractive areas of the city, also into the suburban detached housing areas, keeping levels of ethnic segregation high (Leetmaa *et al*. 2016). Hence, longitudinal studies are need in order to find our whether the established inter‐ethnic contacts survive over the life course.

Not all ethnically mixed places might be mixed in the sense that they include both Estonians’ and Russians’ leisure styles in an integrative way. The social closure based on ethnic background is more common among Estonians (cf. Barwick 2015), and they only tend to establish relationships of trust, mutuality and reciprocity (cf. Ryan 2016) with minorities who are more like themselves. For example:

Levist väljas is one bar where only Russians who speak Estonian go. In that sense it is such a cool place, there they go and are friendly, all of them. When they want to be Estonians then they go there, or something. (Male, 28, Estonian)

In other words, many mixed ethnic places could also be called Estonian places where more integrated or assimilated Russians also tend to go. This example shows that Russian‐speaking people – especially those who are from mixed ethnic families and who are already well integrated – are sometimes in a situation where they can choose, for example, if they feel more like ‘Estonians’ or ‘Russians’. Then they act accordingly when they go out.

This means that places are not necessarily becoming more diverse and mixed, but people with a Russian background become more assimilated into Estonian culture. Assimilation instead of integration is, interestingly, especially expected by younger Estonians, who often classify Russians as being ‘typical’ and ‘non‐typical’, considering ‘non‐typical’ those Russians who speak Estonian, behave as Estonians and are otherwise assimilated into Estonian culture without a hint of Russian origin. Such assimilated Russians are accepted into Estonians’ social groups, while feelings towards ‘typical’ Russians are still often deeply prejudiced. In some cases it seems that even the well‐integrated ‘non‐typical’ Russians agree with such classifications and they avoid ‘typical’ Russians themselves:

When I see that there are some discos in ‘Club Parliament’, those are … well … you feel that this is Russians’ party, and you just do not … well, I do not go there. (Female, 45, Russian)

Many Estonian interviewees have even said that if minorities are proficient in Estonian, they do not classify them as Russians but as Estonians, showing that spoken language is the most important factor for acceptance (cf. Valentine & McDonald 2004). In other words, there are some assimilationist underpinnings to the increase in spending free time together or, in other words, Estonians tend to see integration as a one‐way process rather than a two‐way process (cf. Berry 2005). What is important, though, is that the more assimilated Russians still act as a cross‐ethnic bridging group – although somewhat reluctantly and thus not in an ideal way – since they still have relatives and friends among less integrated Russians as well.

## Discussion of the Main Findings

This paper focused on the leisure activities (segmentation) and activity sites (segregation) of ethnic groups in Tallinn by using a domains framework (van Ham & Tammaru 2016). More specifically, we analysed ethnic differences in the out‐of‐home and out‐of‐work free time activities by relating leisure time activities to home and work. Our first main finding is that the segmentation of leisure time activities has converged as the types of activities of members of minority and majority populations have become similar across almost the full board of activities we studied. However, ethnic segregation during leisure time is still high and members of the minority and majority populations undertake similar activities but at different places and at different times. The fact that activities converge implies that there is a potential for leisure to drive ethnic integration.

We investigated whether leisure activity sites relate to places of home and work. Workplaces are often seen as the most important arenas of social interaction (Strömgren *et al*. 2014; Tomaskovic‐Devey 2014). Our findings show that more attention should be paid to residential neighbourhoods. Although people might not have daily meaningful interactions with their neighbours, the location of their homes strongly shapes where, and with whom, they spend their leisure time. We repeatedly found that people undertake most of their daily free time activities close to their homes. This might be especially true among ethnic minorities who are less likely to be employed than Estonians. Using the terminology of Silm and Ahas (2014), home is still the most important anchor point for people. Hence, leisure could be partly characterised as the ‘long arm of home’.

The case of Estonia might be particularly illustrative. Although Estonia has hardly had any immigration in recent decades, and minority groups have been firmly established for three generations now, the Estonian case shows that it is still difficult to cross ethnic borders in social relations. As Valentine (2008) has argued, inter‐ethnic interaction is always shaped by history, material conditions, and power‐relations. However, the combination of joint interests and a leisure setting which allows less formal social relations and power relations compared to those with co‐workers and neighbours could facilitate inter‐ethnic integration ‘due to the qualities of free choice and self‐determination, which are important because they give individuals the opportunity to freely choose their companions’ (Shinew *et al*. 2004, p. 338).

Our analysis shows that such potential is harnessed only by the most integrated or even assimilated members of the minority population. The social closure based on ethnic background is more common among Estonians, and they tend to establish relationships of trust, mutuality and reciprocity (cf. Ryan 2016) only with minorities who are more like themselves and who are taking part in the leisure activities structured around Estonian values. Even the group of ‘diversity‐seekers’ (cf. Peters & de Haan 2011) are no exception in this regard. Furthermore, the bridging role of assimilated Russians across ethnic lines remains limited since in order to enter into the social networks of Estonians, they have to distance themselves from other minorities.

Hence, in an ethnically diverse city with no major distinctions in the activities that members of the different ethnic groups undertake, the force of homophily and differences in taste (e.g. the milieu of concrete leisure places) still sort different ethnic groups into different activity sites. Our findings show, for example, that when going out to cafés preferences matter. The atmosphere, the choice of music (including the language of songs), the different rules and more subtle cultural codes attached to different places all play a role in the choice process. Ethnic groups thus tend to preserve clear segregation of leisure places, or ‘leisure enclaves’ (Chavez 2000). Even being at the same place at the same time does often not lead to interaction during the out‐of‐home and out‐of‐work leisure activities, since people prefer to talk with in‐group rather than with out‐group members. Especially when the majority language proficiency is modest among members of the minority population, micro‐level segregation is common since people prefer to interact with those with whom they have lower language barriers, allowing them to express themselves more freely (cf. Stodolska 2007).

What seems very important for deeper inter‐ethnic social interaction is that Estonians expect assimilative rather than integrative behaviour by minorities. Estonians assume minorities to become fluent in the Estonian language and start to behave like members of the majority population. Such assimilated minorities are considered to be like Estonians, removing barriers of deeper inter‐ethnic social interaction. This means that achieving equal incomes, working in the same workplaces and living in the same neighbourhoods are not sufficient preconditions for removing ethnic barriers in leisure time activities, especially when it comes to over‐coming segregation during free time activities. We did not find evidence that this works the other way round; Estonians do not tend to assimilate with minority activities. Members of the majority population hardly try to fit in with the norms and language of the minority population, which would lead towards deeper inter‐ethnic contact and interaction. Especially in the younger generations, who are paradoxically in general more tolerant towards others, deeper inter‐ethnic social interactions rely even more on ethnic minorities accepting the norms of Estonian culture and being proficient in the Estonian language.

To conclude, the domains approach to understanding ethnic segregation offers an important framework for understanding segregation during out‐of‐home and out‐of‐work leisure time activities. The approach emphasises the interrelationships between various life spheres, which in the context of leisure time activities are mostly the domains home and work. We find that residential locations strongly structure the places where people go out during their free time. It also appears that it is easier to overcome segmentation (e.g. going to cafés) than segregation (e.g. going to the same cafés at the same time) during leisure time. Similar activities but at different places at different times still showcase the parallel lives of ethnic groups in the urban space. To stimulate integration, policy needs to spend more attention to complex socio‐spatial dimensions of leisure by stimulating the formation of inter‐ethnic places of encounter, and by facilitating true interaction once members of different ethnic groups are at the same place at the same time. Ultimately, important places for such encounters are residential neighbourhoods, and hence a successful integration policy should address residential segregation.

## References

[tesg12307-bib-0001] Amin, A. (2002), Ethnicity and the Multicultural City: Living with Diversity. Environment and Planning A 34, pp. 959–980.

[tesg12307-bib-0002] Barwick, C. (2015), Are Immigrants Really Lacking Social Networking Skills? The Crucial Role of Reciprocity in Building Ethnically Diverse Networks. Sociology 51, pp. 410–428.

[tesg12307-bib-0003] Berry, J.W. (2005), Acculturation: Living Successfully in Two Cultures. International Journal of Intercultural Relations 29, pp. 697–712.

[tesg12307-bib-0004] Jackson E & T. Butler (2014), Revisiting ‘Social Tectonics’: The Middle Classes and Social Mix in Gentrifying Neighbourhoods. Urban Studies 52, pp. 2349–2365.

[tesg12307-bib-0005] Chavez, D.J. (2000), Invite, Include, and Involve! Racial Groups, Ethnic Groups, and Leisure *In*: M.T. Allison & I.E. Schneider , eds. Diversity and the Recreation Profession: Organizational Perspectives. pp. 179–191, State College, PA: Venture Publishing.

[tesg12307-bib-0006] Clayton, J. (2009), Thinking Spatially: Towards an Everyday Understanding of Inter‐ethnic Relations. Social and Cultural Geography 10, pp. 481–498.

[tesg12307-bib-0007] Clayton, J. (2012), Living the Multicultural City: Acceptance, Belonging and Young Identities in the City of Leicester, England. Ethnic and Racial Studies 35, pp. 1673–1693.

[tesg12307-bib-0008] Dixon J. & K. Durrheim (2003), Contact and the Ecology of Racial Division: Some Varieties of Informal Segregation. British Journal of Social Psychology 42, pp. 1–23. 12713753 10.1348/014466603763276090

[tesg12307-bib-0009] Dutton, J.E. , J.M. Duckerich & C.V. Harquail (1994), Organizational Images and Member Identification. Administrative Science Quarterly 39, pp. 239–263.

[tesg12307-bib-0010] Floyd, M and J.H. Gramann (1993), Effects of Acculturation and Structural Assimilation in Resource‐based Recreation: The Case of Mexican Americans. Journal of Leisure Research 25, pp. 6–21.

[tesg12307-bib-0011] Gentin S. (2011), Outdoor Recreation and Ethnicity in Europe – a Review. Urban Forestry and Urban Greening 10, pp. 153–161.

[tesg12307-bib-0012] Harinen, P.M. , M.V. Honkasalo , J.K. Ronkainen & L.E. Suurpää (2012), Multiculturalism and Young People's Leisure Spaces in Finland: Perspectives of Multicultural Youth. Leisure Studies 31, pp. 177–191.

[tesg12307-bib-0013] Kamenik, K , T. Tammaru & O. Toomet (2015), Ethnic Segmentation in Leisure Time Activities in Estonia. Leisure Studies 34, pp. 566–587.

[tesg12307-bib-0014] Kim, J , S.H. Park , E. Malonebeach & J. Heo (2015), Migrating to the East: A Qualitative Investigation of Acculturation and Leisure Activities. Leisure Studies 35, pp. 421–437.

[tesg12307-bib-0015] Kivijärvi, A. (2015), Fragility of Leisure Ties between Ethnic Minority and Majority Youth – an Empirical Case from Finland. Leisure Studies 34, pp. 150–165.

[tesg12307-bib-0016] Leetmaa, K. , T. Tammaru & D.B. Hess (2015), Preferences toward Neighbor Ethnicity and Affluence: Evidence from an Inherited dual Ehnic Context in Post‐Soviet Tartu, Estonia. Annals of the Association of American Geographers 105, pp. 162−182.

[tesg12307-bib-0017] Mägi, K , K. Leetmaa T. Tammaru & M. van Ham (2016), Types of spatial mobility and change in people's ethnic residential contexts. Demographic Research 34, pp. 1161−1192.

[tesg12307-bib-0018] Marcińczak , S, S. Musterd , M. van Ham M & T. Tammaru (2016), Inequality and Rising Levels of socio‐economic Segregation: Lessons from a Pan‐European Comparative Study In: Tammaru T., Marcińczak S, van Ham M & Musterd S, eds., Socio‐economic Segregation in European Capital Cities: East Meets West (pp. 358–378). Abingdon: Routledge.

[tesg12307-bib-0019] McPherson, M. , L. Smith‐Lovin & M. Cook (2001), Birds of a Feather: Homophily in Social Networks. Annual Review of Sociology 27, pp. 415–444.

[tesg12307-bib-0020] Meissner, M. (1971), The Long Arm of the Job: A Study of Work and Leisure. Industrial Relations 10, pp. 239–260.

[tesg12307-bib-0021] Mooses, V. , S. Silm & R. Ahas (2016), Ethnic Segregation During Public and National Holidays: A Study Using Mobile Phone Data. Geografiska Annaler: Series B, Human Geography 98, pp. 205–219.

[tesg12307-bib-0022] Oldenburg, R. (1989), The Great Good Place. New York: Marlowe & Co.

[tesg12307-bib-0023] Peters, K. & H. de Haan (2011), Everyday Spaces of Inter‐ethnic Interaction: The Meaning of Urban Public Spaces in the Netherlands. Leisure 35, pp. 169–190.

[tesg12307-bib-0024] Piekut, A. , P. Rees , G. Valentine & M. Kupiszewski (2012), Multidimensional Diversity in Two European Cities: Thinking beyond Ethnicity. Environment and Planning A 44, pp. 2988–3009.

[tesg12307-bib-0025] Roberts, K. , C. Fagan , I. Boutenko & K. Razlogov (2001). Economic Polarization, Leisure Practices and Policies, and the Quality of Life: A Study in Post‐Communist Moscow. Leisure Studies 20, pp. 161–172.

[tesg12307-bib-0026] Ryan, L. (2016), Looking for Weak Ties: Using a Mixed Methods Approach to Capture Elusive Connections: Looking for Weak Ties. Sociological Review 64, pp. 951–969.

[tesg12307-bib-0027] Schaeffer, M. (2013), Inter‐ethnic Neighbourhood Acquaintances of Migrants and Natives in Germany. On the Brokering Roles of Inter‐ethnic Partners and Children. Journal of Ethnic and Migration Studies 39, pp. 1219–1240.

[tesg12307-bib-0028] Schelling, T.C. (1971), Dynamic Models of Segregation. Journal of Mathematical Sociology 1, pp. 143–186.

[tesg12307-bib-0029] Schrover, M. , J. van der Leun & C. Quispel (2007), Niches, Labour Market Segregation, Ethnicity and Gender. Journal of Ethnic and Migration Studies 33, pp. 529–540.

[tesg12307-bib-0030] Sheller, M. & J. Urry (2006), The New Mobilities Paradigm. Environment and Planning A 38, pp. 207–226.

[tesg12307-bib-0031] Shinew, K.J. , T.D. Glover & D. Parry (2004), Leisure Spaces as Potential Sites for Interracial Interaction: Community Gardens in Urban Areas. Journal of Leisure Research 36, pp. 336–355.

[tesg12307-bib-0032] Silm, S. & R. Ahas (2014), Ethnic Differences in Activity Spaces: A Study of Out‐of‐home Nonemployment Activities with Mobile Phone Data. Annals of the Association of American Geographers 104, pp. 542–559.

[tesg12307-bib-0033] Sime, D. & R. Fox (2014), Migrant Children, Social Capital and Access to Services Post‐migration: Transitions, Negotiations and Complex Agencies. Children & Society 29, pp. 524–534.

[tesg12307-bib-0034] Stodolska, M. (2007), Social Networks, Ethnic Enclosure and Leisure Behavior of Immigrants from Korea, Mexico, and Poland. Leisure 31, pp. 277–324.

[tesg12307-bib-0035] Strömgren, M. , T. Tammaru , A.M. Danzer , M. van Ham , S. Marcinczak , O. Stjernström & U. Lindgren (2014), Factors Shaping Workplace Segregation between Natives and Immigrants. Demography 51, pp. 645–671. 24399142 10.1007/s13524-013-0271-8

[tesg12307-bib-0036] Tammaru, T. , S. Marcińczak , M. van Ham & S. Musterd , eds. (2016), Socio‐economic Segregation in European Capital Cities. East meets West. London: Routledge.

[tesg12307-bib-0037] Toomet, O. , S. Silm , E. Saluveer , T. Tammaru & R. Ahas (2015), Where do Ethnic Groups Meet? Copresence at Places of Residence, Work, and Free‐time. PlosOne 10. doi: 10.1371/journal.pone.0126093. PMC4440775 25996504

[tesg12307-bib-0038] Tomaskovic‐Devey, D. (2014), The Relational Generation of Workplace Inequalities. Social Currents 1, pp. 51–73.

[tesg12307-bib-0039] Valentine, G. (2008), Living with Difference: Reflections on Geographies of Encounter. Progress of Human Geography 32, pp. 323–337.

[tesg12307-bib-0040] Valentine, G. & I. McDonald (2004), Understanding Prejudice: Attitudes towards Minorities. London: Stonewall.

[tesg12307-bib-0041] Valentine, G. & J. Sadgrove (2012), Lived Difference: A Narrative Account of Spatiotemporal Processes of Social Differentiation. Environment and Planning A 44, pp. 2049–2063.

[tesg12307-bib-0042] van Ham, M. & T. Tammaru (2016), New Perspectives on Ethnic Segregation over Time and Space. A Domains Approach. Urban Geography 37, pp. 953–962.

[tesg12307-bib-0043] van Kempen, R. & B. Wissink (2014), Between Places and Flows: Towards a New Agenda for Neighbourhood Research in an Age of Mobility. Geografiska Annaler: Series B Human Geography 96, pp. 95–108.

[tesg12307-bib-0044] Vertovec S. (2007), Super‐diversity and its Implications. Ethnic and Racial Studies 30, pp. 1024–1054.

[tesg12307-bib-0045] Wellman, B. (1996), Are Personal Communities Local? A Dumptarian Reconsideration. Social Networks 18, pp. 347–354.

